# Identifying Cancer-Relevant Mutations in the DLC START Domain Using Evolutionary and Structure-Function Analyses

**DOI:** 10.3390/ijms21218175

**Published:** 2020-10-31

**Authors:** Ashton S. Holub, Renee A. Bouley, Ruben C. Petreaca, Aman Y. Husbands

**Affiliations:** 1 Department of Molecular Genetics, The Ohio State University, Columbus, OH 43215, USA; holub.28@osu.edu; 2 Center for Applied Plant Sciences (CAPS), The Ohio State University, Columbus, OH 43215, USA; 3 Department of Chemistry and Biochemistry, The Ohio State University, Marion, OH 43302, USA; bouley.8@osu.edu; 4 Department of Molecular Genetics, The Ohio State University, Marion, OH 43302, USA; petreaca.1@osu.edu

**Keywords:** evolution, structure-function analysis, ligand-binding domain, tumor suppressor, computational genomics, COSMIC, novel druggable therapeutic targets

## Abstract

Rho GTPase signaling promotes proliferation, invasion, and metastasis in a broad spectrum of cancers. Rho GTPase activity is regulated by the deleted in liver cancer (DLC) family of bona fide tumor suppressors which directly inactivate Rho GTPases by stimulating GTP hydrolysis. In addition to a RhoGAP domain, DLC proteins contain a StAR-related lipid transfer (START) domain. START domains in other organisms bind hydrophobic small molecules and can regulate interacting partners or co-occurring domains through a variety of mechanisms. In the case of DLC proteins, their START domain appears to contribute to tumor suppressive activity. However, the nature of this START-directed mechanism, as well as the identities of relevant functional residues, remain virtually unknown. Using the Catalogue of Somatic Mutations in Cancer (COSMIC) dataset and evolutionary and structure-function analyses, we identify several conserved residues likely to be required for START-directed regulation of DLC-1 and DLC-2 tumor-suppressive capabilities. This pan-cancer analysis shows that conserved residues of both START domains are highly overrepresented in cancer cells from a wide range tissues. Interestingly, in DLC-1 and DLC-2, three of these residues form multiple interactions at the tertiary structural level. Furthermore, mutation of any of these residues is predicted to disrupt interactions and thus destabilize the START domain. As such, these mutations would not have emerged from traditional hotspot scans of COSMIC. We propose that evolutionary and structure-function analyses are an underutilized strategy which could be used to unmask cancer-relevant mutations within COSMIC. Our data also suggest DLC-1 and DLC-2 as high-priority candidates for development of novel therapeutics that target their START domain.

## 1. Introduction

**Figure 1:**
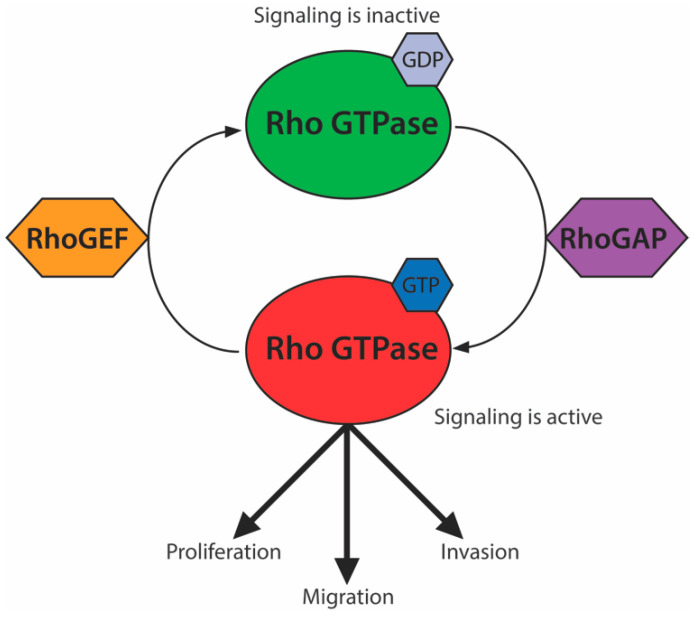
The Rho GTPase signaling module. Rho GTPases (red) are active while bound to GTP. Rho GTPase-activating proteins (RhoGAPs) (purple) stimulate hydrolysis of bound GTP, switching Rho GTPases to an inactive GDP-bound state. Rho guanine nucleotide exchange factors (RhoGEFs) (orange) exchange bound GDP for GTP, reactivating Rho GTPase signaling, which promotes cellular proliferation, migration, and invasion.

Rho GTPases are a subfamily of G proteins involved in signal transduction. These proteins regulate multiple pathways, including the actin cytoskeleton, cell polarity, cell cycle progression, and microtubule dynamics [1]. Signaling is activated by GTP binding at their GTPase domain and is inactivated by its hydrolysis into GDP. This balance is mediated by the opposing activities of Rho guanine nucleotide exchange factors (RhoGEFs) and Rho GTPase-activating proteins (RhoGAPs), which promote GTP replacement and GTP hydrolysis, respectively (Figure 1). Upregulated signaling of Rho GTPases, such as RhoA, Cdc42, and Rac, or their respective RhoGEFs leads to tumor proliferation, migration, invasion, and metastasis in a number of cancers [2,3,4].

Rho GTPases have a globular structure with a limited druggable surface, making them particularly challenging drug targets [5,6,7]. Efforts to mitigate the effects of Rho GTPases in cancer have therefore focused primarily on disrupting aspects of Rho GTPase signaling (reviewed in [7]). These include studies of RhoGAPs, which have specific consistent inhibitory effects on Rho GTPase signaling and are considered particularly attractive components to target within the Rho GTPase signaling module [1,3,5,7,8,9,10,11,12,13,14] (Figure 1). The deleted in liver cancer 1 (DLC-1 or STARD12), DLC-2 (STARD13), and DLC-3 (STARD8) RhoGAPs are frequently down-regulated in cancer and associated with poor prognosis [12,15]. The Cancer Genome Atlas (TCGA) data indicate that of the three DLC genes, DLC-1 is usually the most dramatically down-regulated, followed by DLC-2 [12]. However, unlike DLC-2, DLC-1 is embryonic lethal when knocked out in mouse models [16,17]. A recent pan-cancer analysis also showed that DLC-1 missense mutations are common in cancer cells [15]. This suggests a larger role for DLC-1 in the control of disease and development. DLC-3, on the other hand, does not show a correlation between copy number loss and cancer progression in the TCGA dataset [12]. Despite making differential contributions to cancer progression, all three DLC proteins are considered to be bona fide tumor suppressors that inhibit cancer cell growth [16,18,19,20,21,22,23,24].

Adjacent to the DLC RhoGAP domain is a StAR-related lipid transfer (START) domain. START domains adopt a helix-grip fold structure, forming deep hydrophobic binding pockets that undergo conformational changes upon binding of lipophilic ligands [25,26,27]. START-containing proteins are distributed throughout Eubacteria and Eukaryota and are important regulators of many biological processes. For instance, in humans, START-containing proteins are associated with numerous diseases, including metabolic, oncogenic, and autoimmune disorders [28,29,30]. Structurally, these proteins can be divided into two classes: minimal START proteins and multidomain START-containing proteins which possess a wide variety of additional functional domains [31]. START domains employ a range of complex regulatory mechanisms [19,20,32,33,34]. Of particular interest, START domains in both minimal and multidomain START-containing proteins are able to stimulate regulatory outputs of interacting partners or co-occurring domains in a ligand-dependent manner [32,35]. In the context of DLC proteins, this would be predicted to manifest as START-dependent promotive effects on tumor suppressor activity.

The presence of a START domain suggests DLC proteins may be regulated by a lipophilic ligand, rendering them more easily targetable by natural or synthetic small molecules. However, while there is considerable in vivo and in vitro evidence for them as bona fide tumor suppressors, the role of their START domain remains poorly understood. In fact, the residues mediating START function remain virtually unknown. This is a key bottleneck for targeted modulation of DLC tumor suppressive activity. Here, we use an evolutionary approach to identify conserved residues in DLC START domains that are also mutated in the Catalogue of Somatic Mutations in Cancer (COSMIC) dataset. We find that conserved residues of DLC-1 and DLC-2 START domains are highly overrepresented in tumor samples from a broad spectrum of cancers. Moreover, using comparative structural modeling, we identify a set of three residues, present in both DLC-1 and DLC-2 START domains, that cluster and interact at the tertiary level. Mutations identified in COSMIC are predicted to break these non-covalent interactions and disrupt START tertiary structure and function. Our results support the notion that the START domain is involved in DLC-mediated tumor suppression and that DLC proteins may, thus, be particularly tractable therapeutic targets. Importantly, these residues would not have emerged from traditional scans for mutational hotspots. As such, we propose that tandem evolutionary and structural analyses could help unmask hidden cancer-relevant mutations within the COSMIC dataset.

## 2. Results and Discussion

### 2.1. Mutations in Conserved Residues of DLC-1 and DLC-2 START Domains Are Overrepresented in Tumors

COSMIC contains numerous missense, nonsense, and frameshift mutations in the START domains of DLC-1, DLC-2, and DLC-3 [36]. We focused on missense mutations, as the latter two are more likely to yield strong effects that confound specific contributions of the DLC START domain. We found 47, 33, and 53 missense mutations falling within the DLC-1, DLC-2, and DLC-3 START domains, respectively (Tables S1–S3). To begin to identify potentially clinically-relevant mutations, we utilized two computational predictors of pathogenicity: combined annotation-dependent depletion (CADD) [37] and the rare exome variant ensemble learner (REVEL) [38]. Both tools scored a large number of mutations as potentially disease-causing, with the meta-predictor REVEL returning fewer candidates than CADD, as expected (Tables S1–S3) [38]. To narrow this list of candidates further, we performed a Kolmogorov–Smirnov (K–S) test for uniformity, commonly used to identify mutational hotspots in cancer [39]. As the K–S test failed to reject the null hypothesis, these mutations do not seem to cluster into obvious hotspots at the primary amino acid sequence level (Table S4). Instead, we hypothesized that clustering occurs at the level of functional residues, which are distributed along the length of the DLC START domain.

**Figure 2:**
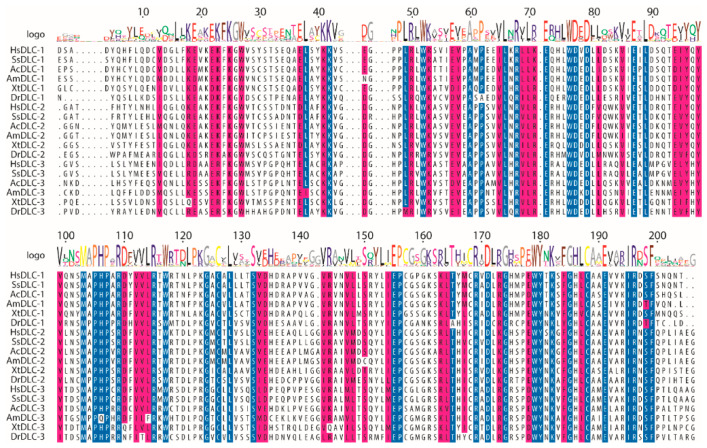
Representative subset of the multiple sequence alignment (MSA) of StAR-related lipid transfer (START) domains from DLC-1, DLC-2, and DLC-3 orthologs. Species include *Homo sapiens* (Hs), *Sus scrofa* (Ss), *Aquila chrysaetos* (Ac), *Alligator mississippiensis* (Am), *Xenopus tropicalis* (Xt), and *Danio rerio* (Dr). Blue indicates residues that are identical in ≥98% of sequences. Magenta indicates residues with similar physiochemical properties in ≥98% of sequences. White represents non-conserved residues. Logo represents the consensus residue(s) of the 123 sequences used in the full alignment (see Figure S1).

**Figure 3:**
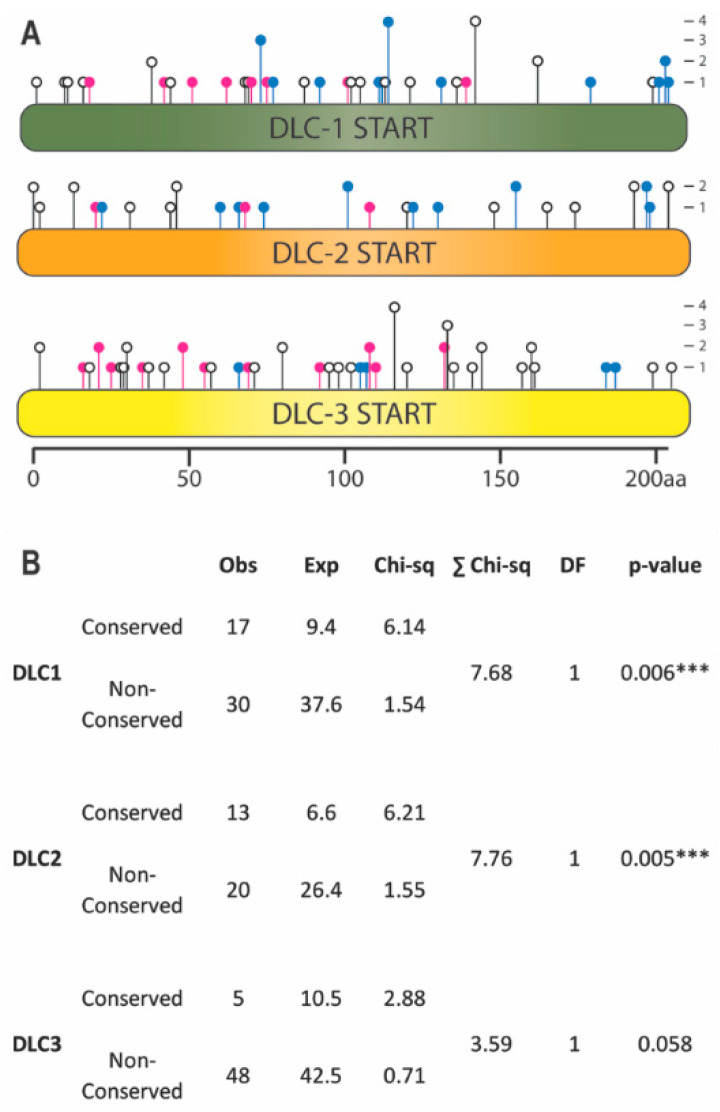
Catalogue of Somatic Mutations in Cancer (COSMIC) missense mutations localizing to conserved residues of DLC-1, DLC-2, and DLC-3 START domains. (**A**) Lollipop plot displaying the location and frequency of missense mutations. Mutations occur in identically conserved (blue), physiochemically conserved (magenta), or non-conserved residues (white). (**B**) COSMIC missense mutations are overrepresented in the evolutionarily-conserved residues of the START domains of DLC-1 and DLC-2 but not DLC-3. Content is identical to Table S7. *** *p* < 0.001, chi-square test.

To identify functional residues, we performed a multiple sequence alignment (MSA), as evolutionary conservation is a strong indicator of critical residues within protein domains [40]. START domains were selected from 123 DLC-1, DLC-2, and DLC-3 orthologs from 46 vertebrate species spanning 450 million years of divergent evolution. Using a stringent 98% threshold, we identified 41 residues (~20%) within the 206-amino acid DLC START domain that are identical in ≥98% of sequences (Figure 2; see Figure S1 for full species MSA). Missense mutations from COSMIC were placed onto the MSA to score whether they preferentially affected these conserved residues (Figure 3A). DLC-1 had 17 mutations falling in 11 conserved residues, DLC-2 had 13 mutations falling in 10 conserved residues, and DLC-3 had 5 mutations falling in 5 conserved residues (Figure 3; Tables S1–S3). This corresponds to 36.2%, 39.3%, and 9.3% of total mutations in DLC-1, DLC-2, and DLC-3 START domains, respectively (Table S5). We next tested whether these frequencies deviate from the approximately 20% likelihood of mutations occurring in conserved residues by chance (Table S5). Indeed, mutations are significantly overrepresented in conserved residues of both DLC-1 (*p*-value = 0.006) and DLC-2 (*p*-value = 0.005) START domains (Figure 3B; Tables S5–S7). By contrast, mutations of the DLC-3 START domain do not appear to be overrepresented in conserved residues (*p*-value = 0.058). These findings are consistent with the frequent downregulation of DLC-1 and DLC-2, but not DLC-3, in many cancers [12]. Taken together, these evolutionary analyses identify conserved residues in DLC-1 and DLC-2 START domains which may be linked to cancer phenotypes.

### 2.2. Conserved Residues form Multiple Bonds within the START Tertiary Structure

Conservation of residues implies critical roles in function [40]. For example, an arginine residue near the opening of the START cavity is highly-conserved and known to be required for START activity [41]; this residue also accrued mutations in multiple independent patient samples (DLC-1 R988; Table S1). Furthermore, mutation of the conserved E966 residue of DLC-1 identified in our analysis impairs tumor suppressor activity [15] (Table S1). Note that this E966K substitution was predicted to be benign by REVEL (Table S1), despite having a clear effect on DLC-1 activity. In addition, a 29-amino acid deletion of the DLC-1 START domain severely reduces Caveolin-1 interaction and DLC-1 tumor suppressor activity [20]. This region contains two conserved residues (R947 and E951) and three residues with evolutionarily-conserved physicochemical properties (E936, I944, L949) (Table S1). The recovery of multiple residues with known roles in either cancer or START activity supports the biological relevance of the residues identified by our analyses. Most conserved DLC START residues are uncharacterized, however, and it is formally possible that amino acid substitutions have minimal effects on tertiary structure [42]. We thus sought to predict their impact on structure and function of DLC START domains using comparative structural modeling. As mutations in the DLC-3 START domain were not overrepresented in COSMIC, it was excluded from these structural analyses.

**Figure 4:**
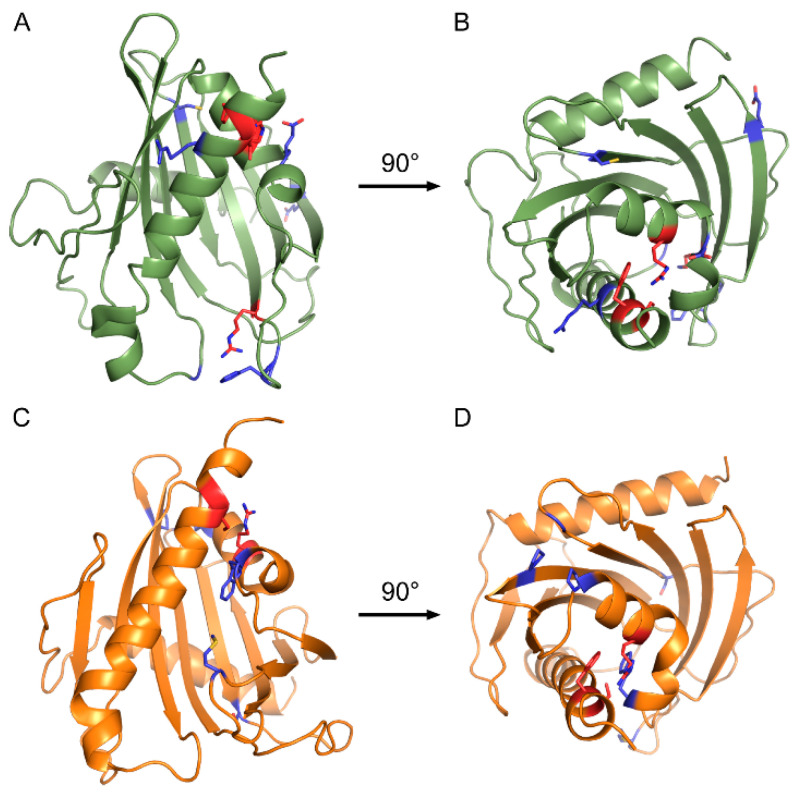
Homology models of DLC-1 and DLC-2 START domains. (**A**,**C**) Side and (**B**,**D**) top-down views of (**A**,**B**) DLC-1 (green) and (**C**,**D**) DLC-2 (orange) START domains. Blue indicates conserved residues with missense mutations in cancers. Red indicates conserved arginine, serine, and phenylalanine residues mutated in both DLC-1 and DLC-2, as well as the highly-conserved arginine in DLC-1 (R988) mutated in numerous cancers.

Informed by the crystal structure of truncated DLC-2 (2PSO; [43]), we generated homology models for both DLC-1 and DLC-2 START domains (Figure 4). B factors indicate several loops in the 2PSO structure are mobile, however we do not expect these disordered loops to affect results. To gauge the quality of our models, we examined interactions for the DLC-1 R988 residue described above, and its DLC-2 analog R1010, using the Crystallographic Object-Oriented Toolkit (CooT; [44]). DLC-1 R988 and DLC-2 R1010 occupy similar positions within their respective models, closely align with the 2PSO structure (RMSD—0.402 Å), and form hydrogen bonds with three residues: P986, R1052, and H1054 of DLC-1 and P1088, K1075, and H1077 of DLC-2 (Figure S2). Furthermore, these residues are either conserved (P986/P1088), have evolutionarily-conserved physicochemical properties (R1052/K1075), or are highly overrepresented in vertebrate DLC-1 and DLC-2 sequences (H1054/H1077) (Figure S1). These data suggest that our models are likely to reflect biologically-relevant conformations of DLC-1 and DLC-2 START domains.

Mutations in COSMIC falling within conserved residues were then mapped to these homology models (Figure 4). Strikingly, three conserved residues with missense mutations in COSMIC colocalized to the same positions within the DLC-1 and DLC-2 START domains: R947, S1077, and F1078 of DLC-1 (Figure 4A,B) and R969, S1100, and F1101 of DLC-2 (Figure 4C,D). The conserved R947/R969 residues are part of helix-2 and are in close proximity to the S1077/S1100 and F1078/F1101 residues, which sit near the end of the C-terminal helix-3. Given the degree of conservation and close proximity in both DLC-1 and DLC-2, we focused our subsequent analyses on these arginine, serine, and phenylalanine residues.

**Figure 5:**
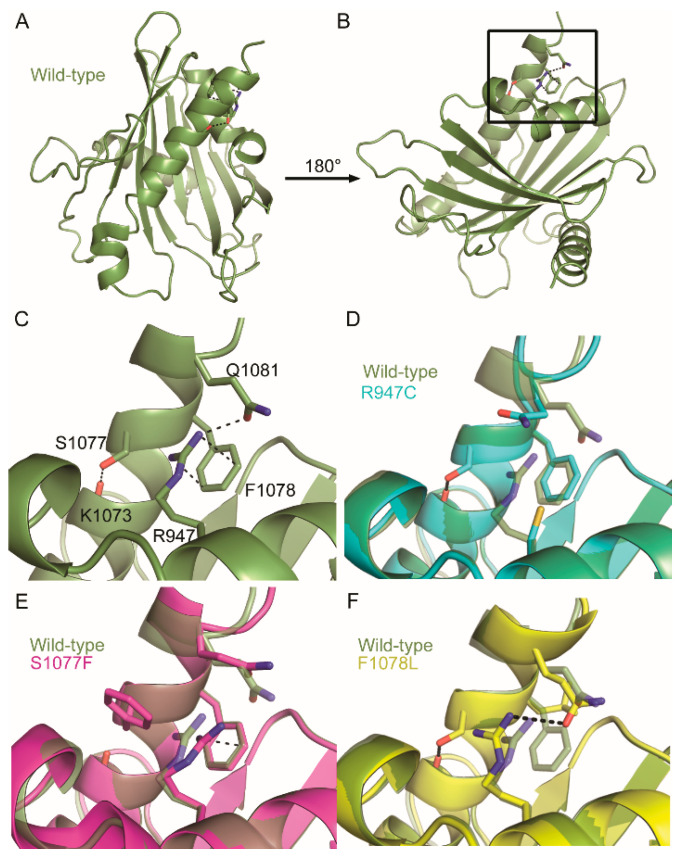
Consequences of COSMIC missense mutations on the tertiary structure of the DLC-1 START domain. (**A**–**C**) Conserved residues colocalizing near the C-terminal helix form multiple tertiary-level interactions in the wildtype DLC-1 START domain (green). (**D**) R947C disrupts the cation–π interaction of F1078 and hydrogen bond of Q1081. (**E**) F1078L disrupts the R947 cation–π interaction but retains the hydrogen bond between R947 and Q1081. (**F**) S1077F retains the cation–π interaction and disrupts all other bonds. Dotted lines indicate hydrogen bonding and cation–π interactions. Structures carrying the R947C, F1078L, and S1077F mutations are blue, pink, and yellow, respectively. (**C**–**F**) are zoom-ins of black box in (**B**).

**Figure 6:**
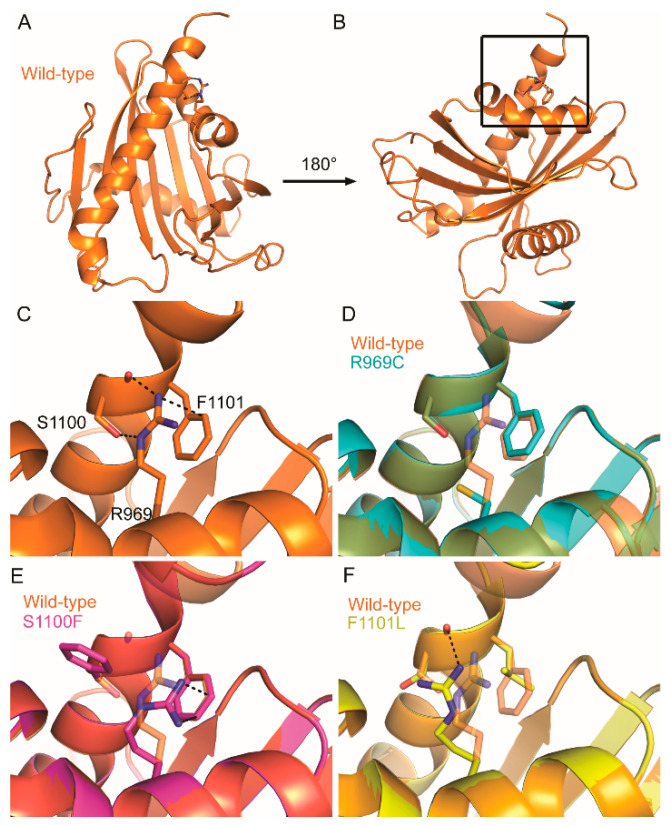
Consequences of COSMIC missense mutations on the tertiary structure of the DLC-2 START domain. (**A**–**C**) Conserved residues colocalizing near the C-terminal helix form multiple tertiary-level interactions in the wildtype DLC-2 START domain (orange). (**D**) R969C mutation disrupts all interactions formed between these residues. (**E**) F1101L disrupts the cation–π interaction of R969 and the hydrogen bond with the side chain of S1100. Dotted lines indicate hydrogen bonding and cation–π interactions. Structures carrying the R969C, S1100F, and F1101L mutations are blue, pink, and yellow, respectively. (**C–F**) are zoom-ins of black box in (**B**).

The close proximity of these residues in three-dimensional space presents the possibility that they may interact at the tertiary level. We examined this idea using CooT [44] and identified multiple predicted interactions between these residues, as well as between these residues and their neighbors. For instance, the R947 residue of DLC-1 forms a cation–π interaction with the aromatic ring of F1078 (3.3 Å; Figure 5A–C). Cation–π interactions in proteins generally occur between a positively charged amino acid and the π face of an aromatic ring and are key contributors to protein structure [45,46]. In addition to this cation–π interaction, R947 also forms a hydrogen bond with Q1081 (3.1 Å). Finally, S1077 does not directly interact with either R947 or F1078 but instead forms a hydrogen bond with K1073 within helix-3. In the case of DLC-2, R969 and F1101 in DLC-2 similarly form a cation–π interaction, although the strength of this interaction is predicted to be weaker (3.8 Å; Figure 6A–C). In contrast to S1077 of DLC-1, S1100 in DLC-2 interacts with R969 via hydrogen bonding at both its carboxy backbone (2.9 Å) and side chain (3.2 Å) of S1100. Thus, while not evident from the primary sequence, three-dimensional homology models reveal that these residues appear to function together at the tertiary level.

### 2.3. COSMIC Missense Mutations Disrupt Tertiary-Level Interactions in DLC-1 and DLC-2 START Domains

Their conservation, proximity, and extensive intermolecular interactions suggest that missense mutants in the arginine, serine, or phenylalanine residues would disrupt START structure and function. To test this, we separately replaced each residue with one of the missense mutations identified in COSMIC (Tables S1–S3). We then generated new homology models for DLC-1 and DLC-2 START domains and used CooT to determine the likely effect of these mutations on the previously identified noncovalent network.

In DLC-1, the R947C mutation abolishes its cation–π interaction with F1078 and hydrogen bond with Q1081 but leaves the S1077–K1073 hydrogen bond intact (Figure 5D). S1077F retains the R947 and F1078 cation–π interaction but abolishes the S1077F–K1073 hydrogen bond, as well as the hydrogen bond between R947 and Q1081 (Figure 5E). Finally, F1078L disrupts its cation–π interaction with R947 but retains the hydrogen bond between R947 and Q1081, as well as the S1077–K1073 hydrogen bond (Figure 5F).

In the case of DLC-2, the R969C mutation similarly abolishes its cation–π interaction with F1101, as well as its hydrogen bond to S1100 (Figure 6D). S1100F retains the cation–π interaction with R969 and F1101 while abolishing all hydrogen bonds formed with R969 (Figure 6E). Finally, F1101L abolishes its cation–π interaction with R969 and disrupts the hydrogen bond between R969 and the side chain of S1100 but retains the hydrogen bond between R969 and the carboxyl backbone of S1100 (Figure 6F).

The arginine-to-cysteine mutations in both DLC-1 (R947C) and DLC-2 (R969C) disrupt cation–π interactions, as well as multiple hydrogen bonds. Thus, we predict this substitution to be the most destabilizing in both START domains. This is also in line with global analyses of the COSMIC dataset, which found that arginine-to-glutamine and arginine-to-cysteine substitutions occur with high frequency in driver mutations [47]. The serine-to-phenylalanine mutations (S1077F in DLC-1 and S1100F in DLC-2) also abolish multiple hydrogen bonds and replace a small polar side chain with a large hydrophobic side chain. Thus, this substitution could conceivably destabilize START domains to a similar extent as the arginine-to-cysteine mutations. By contrast, the phenylalanine-to-leucine mutations (F1078L and F1101L) are predicted to be the least destabilizing, as several hydrogen bonds are left intact and both amino acids have similar physicochemical properties [42]. These structural analyses, focusing on a subset of mutations overrepresented in patient tumors, support a role for the START domain in regulating the tumor suppressor activity of DLC-1 and DLC-2.

## 3. Conclusions

Functional residues mediating the tumor suppressive activity of the DLC START domain remain virtually unknown. Here, we undertook a pan-cancer analysis and identified residues within DLC-1 and DLC-2 START domains likely to affect the function of these genes. We also show that COSMIC missense mutations in DLC-1 and DLC-2 START domains are significantly overrepresented in evolutionarily-conserved residues predicted to be critical for proper folding, interaction, and/or activity [40,41]. Importantly, the relevance of these potentially clinically-relevant residues to START and DLC tumor suppressive activities is supported by multiple previous findings [15,20]. Our analyses thus provide an evolutionary rationale to begin drug targeting of key residues within the conserved START domains of these genes.

Three of these substitutions, occurring in both DLC-1 and DLC-2, disrupt multiple tertiary-level interactions and are likely to impact START structure. The remaining overrepresented COSMIC missense mutations may similarly impact START activity or affect other properties, such as ligand binding or conformational change. A potential DLC START regulatory mechanism is suggested by studies of both minimal and multidomain START-containing proteins, demonstrating that START domains can stimulate regulatory outputs in an allosteric manner [32,35]. If the DLC START domain functions similarly, it could promote RhoGAP tumor suppressor activity, possibly via allosteric regulation of intra- or inter-molecular interactions. As mutations and deletions in the DLC-1 START domain do not seem to impact the ability of DLC-1 to reduce RhoA [15,20], START-mediated effects may be independent of the DLC RhoGAP domain.

These data also suggest that mutation hotspots might not capture the full spectrum of cancer-relevant mutations in COSMIC. For instance, the conserved arginine, serine, and phenylalanine residues in DLC-1 and DLC-2 appear to work together to stabilize START structure (Figure 5 and Figure 6). Single substitutions in any of these residues would have the same structural and functional consequences for DLC START activity, thereby diluting its signal in COSMIC. We propose that, in addition to traditional analyses of mutation frequency, COSMIC should be queried using coupled evolutionary and structure-function analyses. These tandem approaches may help unmask hidden cancer-relevant mutations.

DLC-1 and DLC-2 are frequently down-regulated, as opposed to mutated, in cancer [12,48]. For instance, DLC-1 expression is reduced 10-fold in lung adenocarcinoma, 3-fold in hepatocellular carcinoma, 4-fold in breast cancer, and 3-fold in colon cancer [12]. Stimulating the activity of residual DLC-1 and DLC-2 proteins through an agonistic START ligand could be a means to improve patient outcomes, particularly if used in combination with current therapeutic approaches. Importantly, agonistic START ligands have already been developed. In plants, signaling of the hormone abscisic acid (ABA) is mediated by its minimal START domain receptor [49]. Opabactin, a rationally designed agonistic START ligand, has a seven-fold stronger affinity than the native ligand and increases ABA-signaling outputs in vivo by a factor of 10 [50]. Agonistic ligands designed for the DLC START domain could compensate for reduced outputs and would be relevant for a number of cancer types. Rational design of these agonists would be greatly aided by the identification of natural DLC START ligands, which remain unknown. The DLC-2 START cavity contains more polar side chains than the cavities of cholesterol-binding START domains, and DLC START domains were therefore proposed to bind fatty acid, rather than sterol, ligands [43]. Supporting this, the STARD14 START domain, which shares characteristics of the DLC START binding pocket, was recently shown to bind several species of fatty acids [32]. Taken together, our data identify several conserved residues likely to underlie the START-directed regulation of DLC-1 and DLC-2 tumor-suppressive capabilities. DLC-1 and DLC-2 may, thus, be high-priority candidates for development of novel therapeutics targeting their START domain.

## 4. Materials and Methods

### 4.1. Mutation Identification and Kolmogorov-Smirnov Test of Uniformity

Mutation data were obtained from the COSMIC database (v91 GRCh38; https://cancer.sanger.ac.uk/cosmic) for the dominant isoforms of DLC-1 (DLC1_ENST00000358919.6), DLC-2 (ENST00000336934.9), and DLC-3 (ENST00000374599.7). DLC START domains were identified using HHpred [51]: DLC-1 (880aa–1083aa), DLC-2 (903aa–1108aa), and DLC-3 (893aa–1098aa). Mutations outside of the START domain, as well as nonsense, frameshift, and intronic mutations within the START domain, were excluded from our analyses. To ensure only independent mutation events were measured, duplicate samples from the same patient were removed before analysis. Pathogenicity scores were obtained from REVEL (from dbNSFP v.4.0a) [38] and CADD (v1.6) [37] using the ENSEMBLE interface (v. 94; https://www.ensembl.org/Tools/VEP) [52]. Missense mutations were counted for individual residues and a one-sample nonparametric test was done using the Kolmogorov–Smirnov test in IBM SPSS Statistics 26. The observed distribution of missense mutations was tested against the null hypothesis that mutations are uniformly distributed along the sample data. Test option settings were set to a significance value of 0.05, a confidence value of 95%, and to exclude cases test-by-test. User-Missing Values were set to “Exclude”.

### 4.2. Multiple Sequence Alignment (MSA)

To build an MSA and identify conserved residues in DLC START domains, orthologs of DLC-1, DLC-2, and DLC-3 were identified using NCBI’s Blastp program against the reference proteins (refseq_protein) database [53]. In total, 123 full-length sequences were found from 46 vertebrate species. Sequences were aligned using the ClustalW algorithm [54] in the MEGA X software (v.10.1.7; https://www.megasoftware.net/) [55] and trimmed to exclude residues falling outside the START domain. Conservation of residues was analyzed using R and the “msa” package from Bioconductor [56]. A stringent 98% consensus threshold was used, allowing for only 2 of the 123 sequences to contain a non-similar residue at a given position. This threshold was selected to account for rare lineage specific mutations that may be permissive to changes in critical residues but not representative of most sequences [57]. Residues were then shaded according to similarity and a consensus logo was added using R and the “msa” package from Bioconductor. Finally, this file was exported in .tex format and compiled using TeXworks (v.0.6.5; Free Software Foundation, Inc. Boston, MA, USA; Figure S1).

### 4.3. Mutation Counts and Chi-Square Analysis

The expected number of mutations in conserved residues assuming a random distribution was calculated by using the percentage of identical residues multiplied by the total number of mutations in the START domain of each paralog.

The expected number of mutations in non-conserved residues was determined by subtracting the expected number of mutations in conserved residues from the total number of mutations. Chi-square values were calculated using the formula (Equations (1) and (2)):

(1) $$Expected\;mutations\;in\;conserved\;residues\;\;=\;\#\;of\;mutations\;\times \;\frac{\#\;of\;conserved\;residues}{Domain\;length}$$

(2) $$Chi\;square\;value=\;\frac{{\left(Expected-Observed\right)}^{2}}{Expected}$$

These summed chi-square values were then calculated using IBM SPSS Statistics 26.

### 4.4. Homology Modeling and Identification of Tertiary-Level Interactions

Homology modeling of each START domain was performed using the online Max Planck Institute Bioinformatics Toolkit (https://toolkit.tuebingen.mpg.de/) [51]. START domain sequences of DLC-1 and DLC-2 were separately queried using HHpred against the Protein Data Bank (PDB_mmCIF70_20_May) structural database to identify related protein structures for homology modelling. The top 10 structures were identical for both START domains and included the crystal structure of DLC-2 (2PSO). These 10 protein structures (chain A of 2PSO, chain A 2R55, chain A of 2MOU, chain A of 5I9J, chain A of 6L1M, chain A of 2E3P, chain C of 3P0L, chain A of 1LN1, chain B of 3FO5, and chain B of 3QSZ) were forwarded to HHpred-TemplateSelection to generate the MSA. Template alignment was subsequently forwarded to MODELLER [58] to predict the tertiary structures of the DLC-1 and DLC-2 START domains. Structural models were examined using the Crystallographic Object-Oriented Toolkit (v.0.8.7; https://www2.mrc-lmb.cam.ac.uk/personal/pemsley/coot/) to identify predicted interactions formed with the conserved residues using the Environmental Distances tool with a cut-off of 4.0 Å [44]. Mutated DLC-1 and DLC-2 START domains were modeled using the same template alignment as their respective wild-type models and analyzed for predicted interactions in CooT using the same cut-off value.

## Supplementary Materials

The following are available online at https://www.mdpi.com/1422-0067/21/21/8175/s1, Figure S1: Full multiple sequence alignment (MSA) of START domains from DLC-1, DLC-2, and DLC-3 orthologs. Figure S2: DLC-1 R988 and DLC-2 R1010 residues and interactions. Tables S1–S7 in Excel.
