# Supplementary material for: Identifying Cancer-Relevant Mutations in the DLC START Domain Using Evolutionary and Structure-Function Analyses
Source: Int J Mol Sci. 2020 Oct 31;21(21):8175. doi: 10.3390/ijms21218175 (PMC7662654; doi:10.3390/ijms21218175)
Supplement: Supplementary file 1 [file ijms-21-08175-s001.zip › Holub et al 2020, Supp Info Zip/ijms-956968-supp.docx]

**Supplemental Figure and Table Legends**

**Figure S1.** Full multiple sequence alignment (MSA) of START domains from DLC-1, DLC-2, and DLC-3 orthologs. Species are listed down the left side of the MSA. Blue indicates residues that are identical in ≥98% of sequences. Magenta indicates residues with similar physiochemical properties in ≥98% of sequences. White represents non-conserved residues. Logo represents the consensus residue(s) of all 123 sequences.

**Figure S2**. DLC-1 R988 and DLC-2 R1010 residues and interactions. DLC-1 R988 forms hydrogen bonds with P986 (3.7 Å), R1052 (3.2 Å), and H1054 (3.5 Å). DLC-2 R1010 forms hydrogen bonds with P1008 (3.1 Å) and H1077 (3.5 Å).

**Table S1** – COSMIC missense mutations and sequence conservation for DLC-1. Conserved and non-conserved residues in the DLC-1 START domain mutated in COSMIC. Features include: mutation identity (HGVS notation), residue number, number of missense mutations, identity of introduced substitution, level of conservation in MSA, and pathogenicity scores.

**Table S2** – COSMIC missense mutations and sequence conservation for DLC-2. Conserved and non-conserved residues in the DLC-2 START domain mutated in COSMIC. Features include: mutation identity (HGVS notation), residue number, number of missense mutations, identity of introduced substitution, level of conservation in MSA, and pathogenicity scores.

**Table S3** - COSMIC missense COSMIC missense mutations and sequence conservation for DLC-3. Conserved and non-conserved residues in the DLC-3 START domain mutated in COSMIC. Features include: mutation identity (HGVS notation), residue number, number of missense mutations, identity of introduced substitution, level of conservation in MSA, and pathogenicity scores.

**Table S4** - Kolmogorov-Smirnov (K-S) test of uniformity for DLC-1, DLC-2, and DLC-3 START domains. K-S test indicates no deviation from uniformity for all DLC START domains. *P* > 0.05.

**Table S5** – Summary of DLC-1, DLC-2, and DLC-3 mutations in conserved and non-conserved residues. Percentage of residues ≥98% conserved in all DLC START domains (green), percentage of conserved residues with at least one mutation (pink), percentage of all mutations falling in conserved residues (grey).

**Table S6** – Chi-square input value determination. Calculation of Expected numbers of mutations falling in conserved or non-conserved residues (for chi-square test).

**Table S7** – Chi-square analysis. Chi-square tested demonstrating DLC-1 and DLC-2 START domains accrue significantly more mutations in conserved residues than expected by chance. DLC-3 does not show this effect. ****P* < 0.001.
