# Supplementary material for: Identifying Cancer-Relevant Mutations in the DLC START Domain Using Evolutionary and Structure-Function Analyses
Source: Int J Mol Sci. 2020 Oct 31;21(21):8175. doi: 10.3390/ijms21218175 (PMC7662654; doi:10.3390/ijms21218175)
Supplement: Supplementary file 1 [file ijms-21-08175-s001.zip › Holub et al 2020, Supp Info Zip/Supplemental Figures.pdf]

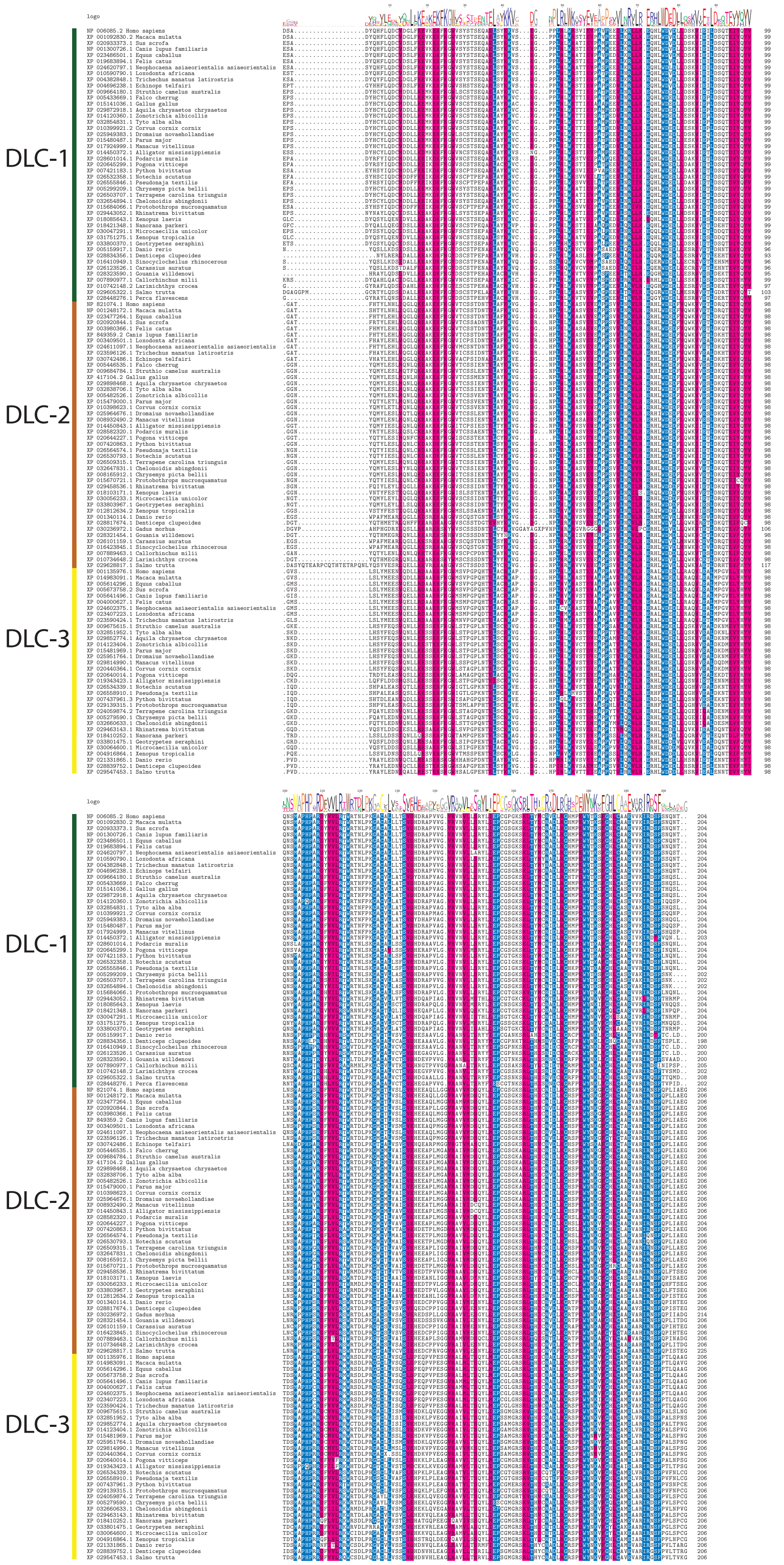

Supp Figure 1. Full multiple sequence alignment (MSA) of START domains from DLC-1, DLC-2, and DLC-3 orthologs. Species are listed down the left side of the MSA.Blue indicates residues that are identical in ≥98% of sequences. Magenta indicates residues with similar physiochemical properties in ≥98% of sequences. White represents non-conserved residues. Logo represents the consensus residue(s) of all 123 sequences.

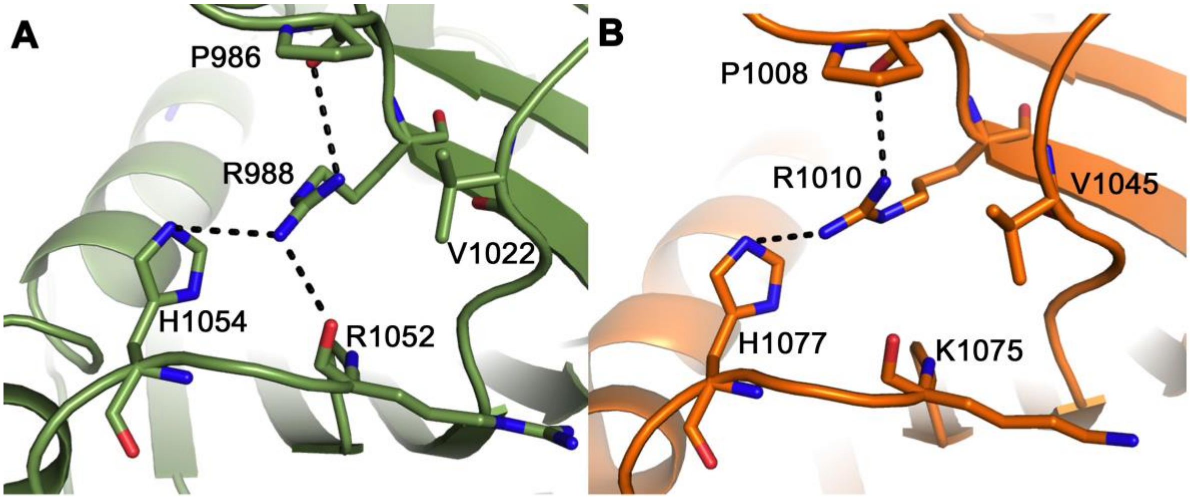

Figure S2. DLC-1 R988 and DLC-2 R1010 residues and interactions. DLC-1 R988 forms hydrogen bonds with P986 (3.7 Å), R1052 (3.2 Å), and H1054 (3.5 Å). DLC-2 R1010 forms hydrogen bonds with P1008 (3.1 Å) and H1077 (3.5 Å).
